# Incessant palpitations in a young male

**DOI:** 10.1002/joa3.12414

**Published:** 2020-08-02

**Authors:** Gregory P. Siroky, Hieu Huynh, Devendra Bisht, Ameesh M. Isath, Davendra Mehta

**Affiliations:** ^1^ Division of Electrophysiology Department of Cardiology Mount Sinai Morningside Icahn School of Medicine at Mount Sinai New York NY USA; ^2^ Department of Medicine Mount Sinai Morningside and West Icahn School of Medicine at Mount Sinai New York NY USA

**Keywords:** intracardiac electrograms, long RP tachycardia, permanent junctional reciprocating tachycardia, supraventricular tachycardia

## Abstract

27‐year‐old male with incessant palpitations and reduced ejection fraction presents for diagnostic electrophysiology study. ECG shows a long RP tachycardia. Permanent junctional reciprocating tachycardia is diagnosed on EP study with successful ablation of posteroseptal accessory pathway.

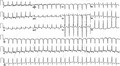

## 1 CASE

**FIGURE 1:**
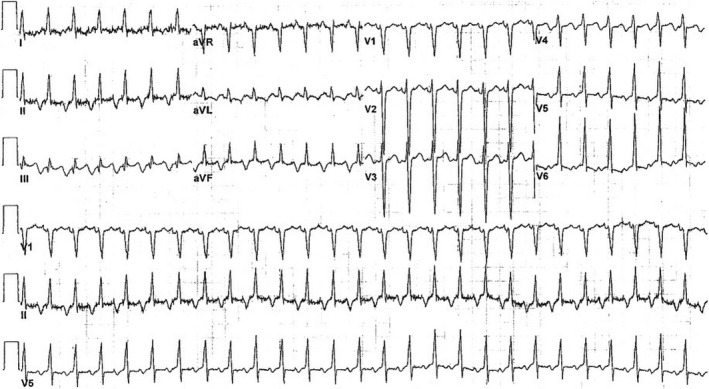
Presenting ECG showing a narrow complex, long RP tachycardia.

A 27‐year‐old male with incessant palpitations and worsening dyspnea on exertion presents for a diagnostic electrophysiology study. His 12‐lead electrocardiogram (ECG) showed a narrow complex, long RP tachycardia as shown in Figure 1. The patient has a long history of palpitations and echocardiogram demonstrated an ejection fraction (EF) of 10%‐15%. What is the differential diagnosis for his tachycardia? What pitfalls can occur when evaluating a young patient with this tachycardia? What is the mechanism of his tachycardia?

## 2 DISCUSSION

The differential diagnosis for a narrow complex, long RP tachycardia includes sinus tachycardia, ectopic atrial tachycardia, an atypical, fast‐slow atrioventricular nodal reentrant tachycardia (AVNRT), or an orthodromic reciprocating tachycardia (ORT) using a decremental, slowly conducting accessory pathway (AP). Close inspection of the patient’s presenting ECG (Figure 1) demonstrates negative P‐waves in the inferior leads and positive P‐waves in lead V1 indicating an inferior and posterior atrial focus. The ECG computer analyzed the rhythm as sinus tachycardia, which can be one of the pitfalls of evaluating a tachycardia of 162 bpm in a young patient whose maximum predicted heart rate (220‐age) is 193 bpm. The P‐wave axis clearly rules out sinus tachycardia as there would be positive P‐waves in the inferior leads and biphasic P‐waves in lead V1.

**FIGURE 2:**
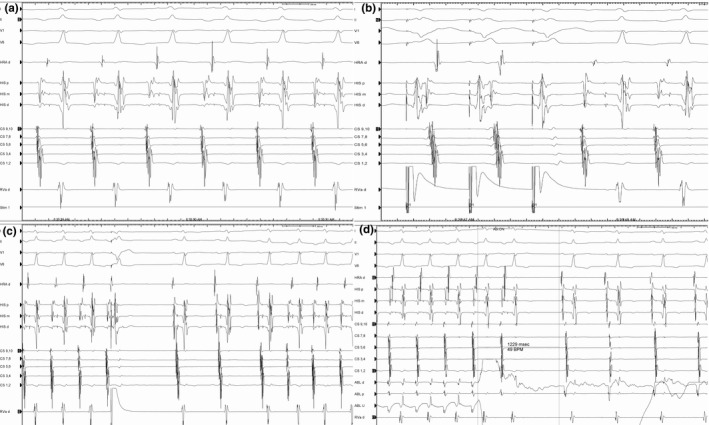
Intracardiac electrograms during the patient’s electrophysiology study. A, Electrograms of the patient’s tachycardia demonstrating a narrow complex, 1:1 atrial‐to‐ventricular ratio tachycardia with the earliest atrial activation in the proximal poles of the coronary sinus catheter. B, Electrograms demonstrating induction of the tachycardia with right ventricular pacing. C, Electrograms demonstrating response of the tachycardia to a His‐refractory premature ventricular depolarization as well as spontaneous re‐initiation of the tachycardia after three sinus beats. D, Termination of tachycardia within 1.2 seconds of initiation of ablation. HRA, high right atrium; HIS p/m/d, proximal, mid, and distal His bundle catheter electrodes; CS 9,10, proximal most electrodes on the coronary sinus (CS) catheter; CS 7,8, second most proximal electrode on the CS catheter; CS 5,6, middle electrodes on the CS catheter; CS 3, 4, second most distal electrodes on the CS catheter; CS 1,2, distal most electrodes on the CS catheter; RVA, right ventricular apex; ABL d, distal ablation catheter electrode; ABL p, proximal ablation catheter electrode, ABL U, unipolar ablation catheter recording.

The patient was brought to the electrophysiology lab for diagnosis and treatment. Upon insertion of the diagnostic catheters, the patient’s tachycardia is demonstrated in Figure 2A, which shows a narrow complex, 1:1 atrial‐to‐ventricular ratio tachycardia with the earliest atrial activation in the proximal poles of the coronary sinus (CS) catheter. Further, pacing maneuvers are demonstrated in Figures 2B,C. In Figure 2B, pacing from the right ventricular apex shows concentric ventriculoatrial (VA) conduction (earliest atrial activation in the proximal His catheter) for the first two beats; however, the third paced complex shows a sudden increase in the VA interval associated with a change in atrial activation (earliest atrial activation is in the proximal CS catheter) and initiation of the clinical tachycardia. Given the ventricular‐atrial‐His‐ventricular (VAHV) response upon cessation of pacing, this excludes an atrial tachycardia. Furthermore, upon cessation of ventricular pacing, the difference between the postpacing interval (515 milliseconds) and the tachycardia cycle length (415 milliseconds) is less than 115 milliseconds making ORT a more likely diagnosis. To further elucidate the diagnosis, a His‐refractory premature ventricular depolarization (PVD) was given as shown in Figure 2C. The His‐refractory PVD terminates the tachycardia without atrial capture demonstrating the presence of an AP as well as its participation in the tachycardia, thereby confirming the diagnosis of ORT. In addition, because of the incessant nature of the clinical tachycardia, there was spontaneous re‐initiation after three sinus beats.

Delving further into the patient’s history, he was diagnosed with fetal supraventricular tachycardia (SVT) in utero necessitating medical therapy and has suffered with palpitations for many years, which was resistant to multiple anti‐arrhythmic medications (digoxin, flecainide, and propranolol). Because of his incessant tachycardia and loss to follow‐up, he sustained tachycardia‐induced cardiomyopathy with an ejection fraction (EF) of 15%‐20%. Given the electrophysiology study and his long history of tachycardia, the most likely diagnosis is permanent junctional reciprocating tachycardia (PJRT), which is an ORT using a retrograde, slowly conducting, decremental AP located at the posteroseptal region of the AV junction. Electroanatomical mapping demonstrated the earliest atrial activation at the 5 o’clock position on the tricuspid annulus as seen from the left anterior oblique (LAO) projection (Supplementary Figure) with subsequent ablation of a right posteroseptal AP. Figure 2D shows successful termination of the tachycardia within 1.2 seconds of initiating ablation. Three months following ablation, the patient’s EF recovered to normal. This case demonstrates the importance of careful examination of an ECG and that despite a relatively older age at presentation, PJRT should remain on the differential diagnosis in a patient who presents with a long RP tachycardia and reduced EF.

## CONFLICT OF INTEREST

Authors declare no conflict of interests for this article.

## Supporting information

Fig S1

