# Supplementary figures and images for: Incessant palpitations in a young male
Source: J Arrhythm. 2020 Aug 2;36(5):945–7. doi: 10.1002/joa3.12414 (PMC7532283; doi:10.1002/joa3.12414)

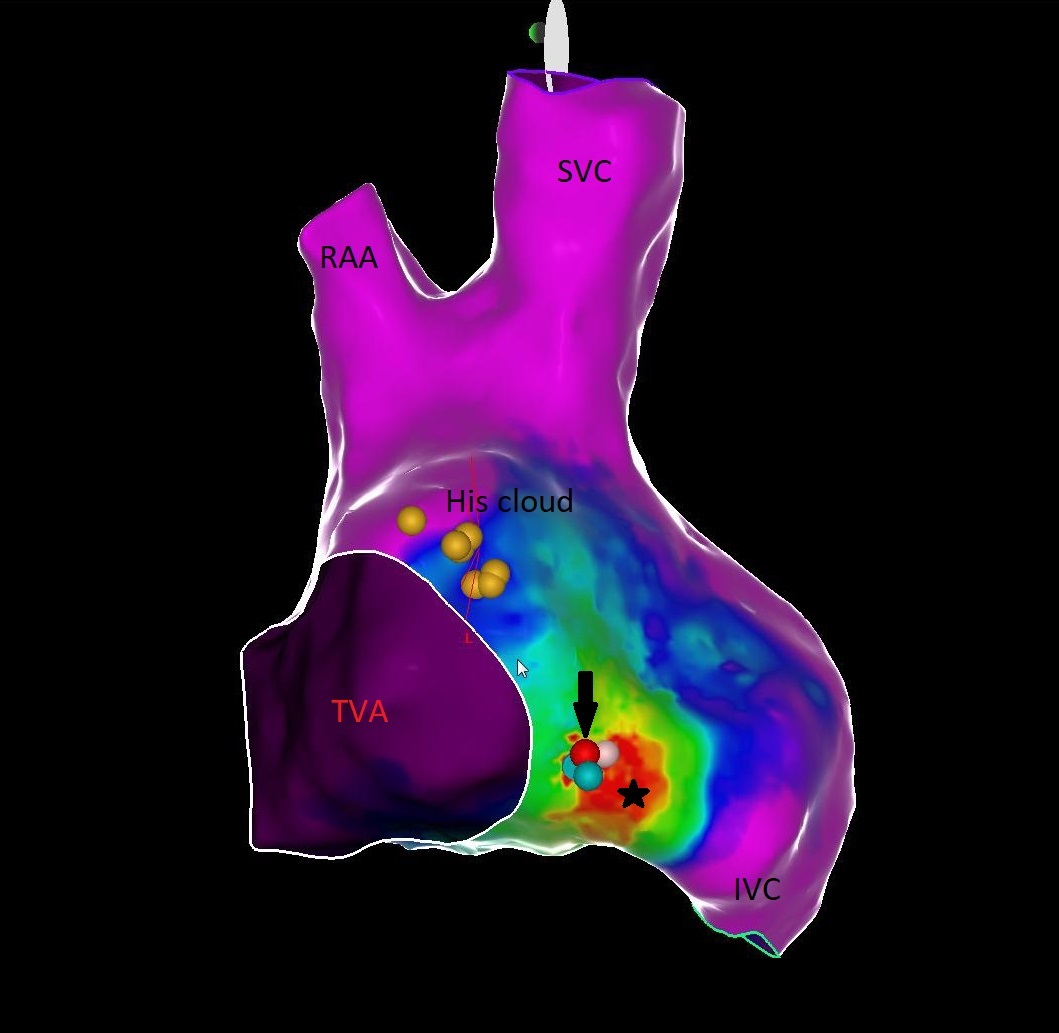

Supplement: Supplementary file 1 — Fig S1 [file JOA3-36-945-s001.jpg]
